# Spread of Extensively Drug-resistant Tuberculosis

**DOI:** 10.3201/eid1304.061329

**Published:** 2007-04

**Authors:** Sofía Samper, Carlos Martín

**Affiliations:** * Hospital Universitario Miguel Servet, Instituto Aragonés de Ciencias de la Salud, Zaragoza, Spain; † University of Zaragoza, Zaragoza, Spain

**Keywords:** Tuberculosis, multidrug resistance, XDR TB, Mycobacterium bovis, letter

**To the Editor:** An emergency has been declared in KwaZulu Natal, South Africa, where an outbreak of 53 cases of a highly lethal form of tuberculosis (TB) has occurred (*1*,*2*). This outbreak was caused by an extensively drug-resistant TB (XDR TB) strain.

XDR TB is defined as TB caused by *Mycobacterium tuberculosis* isolates resistant to isoniazid and rifampicin plus any fluoroquinolone and ≥1 of the 3 injectable second-line drugs (*3*). XDR TB may be considered an emerging disease but not a new disease. Nosocomial outbreaks of multidrug-resistant TB (MDR TB) occurred in Spain at the height of the HIV epidemic, when 49 TB cases were reported in an HIV ward in Madrid from 1991 through 1995 (*4*,*5*). Molecular epidemiology found that a particular strain caused 16 cases in another hospital in Madrid in 1993–1995 (*6*) and 31 cases in a hospital in Malaga in 1995–1998 (*7*,*8*). In total, 22 hospitals from 6 different regions of Spain were affected by this outbreak, which included at least 114 cases, caused by an *M. bovis* XDR strain (B strain) belonging to the *M. tuberculosis* complex. The patients included 1 from the Netherlands (*8*) and another from Canada (*9*).

The strain responsible for the 1991–1995 outbreak in Spain fits the XDR TB case definition; it was resistant to the 5 first-line drugs, as well as to ofloxacin, aminosalicylic acid, cycloserine, ethionamide, capreomycin, amikacin, and clarithromycin. Isolates were tested for drug susceptibility by the Canetti method on Lowenstein-Jensen medium supplemented with isoniazid, rifampicin, ethambutol, streptomycin, amikacin, and pyrazinamide (*6*). The isolates were also tested on 7H10 Middlebrook agar for susceptibility to aminosalicylic acid, ethionamide, capreomycin, clarithromycin, and ofloxacin (*6*). No effective medical treatment was available for these patients. In 2 of the hospitals affected, all patients died, with a short survival time (median of 44 and 49.5 days for the 2 hospitals) between diagnosis and death (*6*,*7*). A high rate of reinfection (45%) also was noted among HIV-positive patients treated with anti-TB drugs (*7*). As a result of this outbreak, Spanish hospitals now implement exhaustive control measures, such as maintaining respiratory isolation units under negative pressure; in addition, a national surveillance network for MDR TB was set up in Spain in 1998. From 1998 through 2003, we detected 22 new cases of infection with this strain (*10*), but no new cases have since been reported to the national MDR TB database.

Our experience indicates that the implementation of more stringent control measures and the use of new, more effective treatments for HIV infection can help to bring XDR TB outbreaks under control in developed countries. However, the outlook is bleak for developing countries like South Africa, in which coinfection with HIV and a highly transmissible and untreatable XDR TB strain could amplify the TB problem to levels unprecedented since the advent of antimicrobial drugs. These countries urgently require assistance with the establishment of control measures and the development of new drugs and effective vaccines against TB.
